# Neuropsychological assessment in preclinical and prodromal Alzheimer disease: a global perspective

**DOI:** 10.7189/jogh.09.010317

**Published:** 2019-06

**Authors:** Tamlyn Watermeyer, Clara Calia

**Affiliations:** 1 Edinburgh Dementia Prevention, Centre for Clinical Brain Sciences, University of Edinburgh, Edinburgh, UK; 2 School of Health in Social Science, University of Edinburgh, Edinburgh, UK; 3 Global Prevention of Dementia Programme (GloDePP), Centre for Global Health Research, University of Edinburgh, UK

Alzheimer disease (AD), the most prevalent form of dementia, refers to a syndrome in which cognitive ability declines to such a degree that functioning in daily and/or social activities is compromised. In 2015, there were approximately 47 million people living with dementia globally, a figure expected to rise to 131 million by 2050. By as soon as 2030, the worldwide prevalence of dementia is estimated to reach 75 million with the majority of cases concentrated in low- and middle-income countries (LMiCs) [1-3]. Ageing worldwide populations beget greater numbers of older individuals living with chronic health conditions, such as dementia, which might pose significant social and economic challenges, most notably, for LMiCs [4]. In high income countries (HiCs), the focus of dementia detection is evolving to further encompass earlier stages of disease with the view to promote future approaches in secondary prevention. It is now accepted that AD-related pathology, such as amyloid and/or tau deposition, occurs decades before the onset of dementia symptoms [5]. The earliest sites of amyloidosis and tauopathy are the medial temporal lobe (MTL) structures, namely the hippocampus and sub-hippocampal areas, followed by neuronal lesions to the neocortical areas [6,7]. Current research nomenclature for AD includes preclinical AD, the earliest stage of AD in which biomarkers, such as amyloid and tau, are detectable through imaging data or cerebrospinal fluid (CSF), but no overt cognitive symptoms are indicated. Preclinical AD precedes another stage, termed prodromal AD where obvious cognitive symptoms emerge alongside aggregating neuropathological change. Finally, continuous neuropathological and cognitive changes culminate in clinical dementia towards the end of the AD continuum (see **Figure 1** and [8]). Different research frameworks for AD have been proposed [9,10] (and references s11 and s12 in **Online Supplementary Document**), but the most recent criteria (ref. s12 in **Online Supplementary Document**) draw the strongest distinction between the biological construct of AD and the corresponding cognitive impairment or dementia syndromes that can accompany underlying disease changes as they evolve. This conceptual shift towards biomarker definitions of AD, away from its related cognitive syndromes, presents significant financial and logistical challenges for low-resourced research and clinical settings in LMiCs where disparate access to infrastructure, equipment and technical expertise required for biomarker capture exist. Consequently, LMiCs risk being excluded from participating in growing global efforts surrounding observational and interventional trials where biological criteria for AD are specified. Here, we consider possible approaches supported by neuropsychology and cognitive neuroscience to circumnavigate some of these challenges in the detection and tracking of AD in LMiC regions for research purposes.

**Figure 1:**
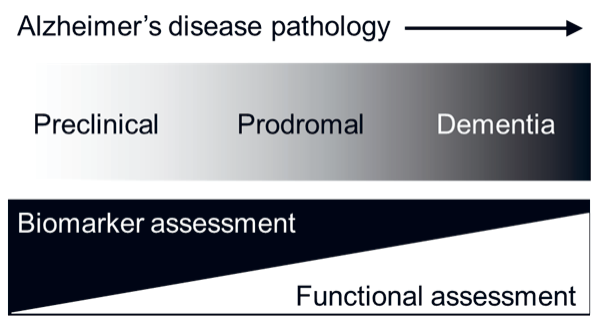
The continuum of Alzheimer disease. The continuum of Alzheimer disease pathology from the preclinical and prodromal stages to overt clinical dementia adapted with permission from [8].

## THE CHANGING POSITION OF NEUROPSYCHOLOGY WITHIN THE LANDSCAPE OF ALZHEIMER DISEASE RESEARCH

**Figure:**
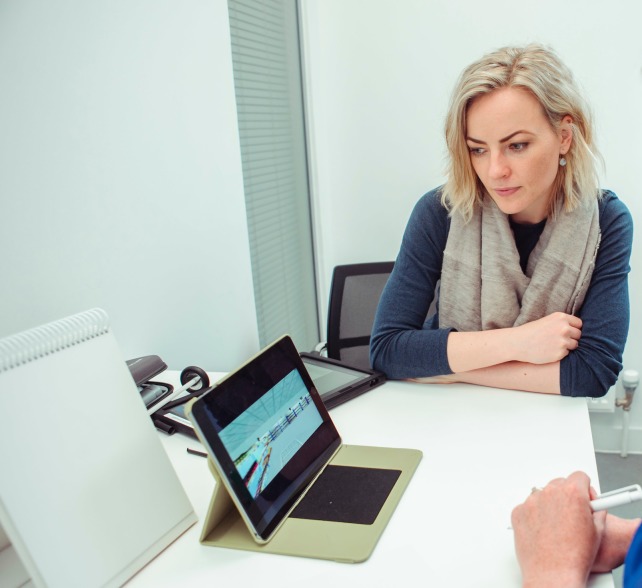
Photo: Example demonstration of the Virtual Supermarket Test being performed on tablet computer (from the author’s research group collection, used with permission).

With an effective therapeutic agent still elusive, research and clinical development efforts are being re-directed towards the preclinical and prodromal stages of disease in order to establish risk factors for AD, model its disease trajectory and identify novel targets for its intervention. These stages after all, may represent the earliest juncture upon which preventative strategies take greatest effect. Pharmaceutical strategies focus on modifying underlying disease agents, such as amyloid and tau, and as a result emphasis is turning towards biomarkers over cognitive markers in the detection and monitoring of early stage disease. However, modification of biological agents through drug interventions has so far been unsuccessful, and in some cases contradictory (ref. s13 in **Online Supplementary Document**), undermining their status as primary indices of therapeutic effect. Furthermore, the prognostic value of these biomarkers remains controversial. Recently, a study evaluating the accuracy of CSF, MRI, genetic and cognitive variables in predicting which middle-aged participants developed symptoms consistent with prodromal AD found that amyloid accumulation and APOE4 status was a less accurate model of participants’ outcome at a five-year follow-up when compared to a model comprising APOE4 status and two cognitive tests. When MRI and CSF phosphorylated tau levels were added to the latter model they provided little improvement in predictive power (ref. s14 in **Online Supplementary Document**). Such findings suggest that outcomes reflecting cognitive function might be relatively more reliable, less invasive and, notably, less costly, indicators of disease. Neuropsychology should therefore focus on refining existing tests and devising novel sensitive measures that map cognitive signals onto AD-specific neuropathological change (ref. s15 in **Online Supplementary Document**). This could be achieved through exploiting growing knowledge from the cognitive neurosciences regarding connections between specific cognitive functions and neural mechanisms that are vulnerable to the earliest pathological insults (ref. s16 in **Online Supplementary Document**). In doing so, sensitive cognitive measures – or combinations thereof – can gain parity of reputation with biomarkers which are increasingly becoming considered the “gold standard” of research diagnosis, despite their aforementioned caveats.

Progress in this area is already under way. Some frameworks of AD propose further staging of the preclinical AD category whereby subtle cognitive decline is detectable alongside biomarker evidence [9]. Attempts to identify cognitive markers for this stage have provided inconsistent findings. Nonetheless, several promising neuropsychological measures have been recommended for research trials of preclinical AD due to their association with amyloid positivity and/or correlation with structural or functional changes in the MTL (ref. s17 and s18 in **Online Supplementary Document**). In addition, various studies have adopted the use of composite scores – indices that combine performance from several cognitive tests into one measure – with the aim of enhancing the sensitivity of traditional measures designed for later stages of AD to detect and track cognitive decline over time (ref. s19 in **Online Supplementary Document**). There have been calls to harmonize protocols of neuropsychological assessment for neurodegenerative conditions across European trials (ref. s20 and s21 in **Online Supplementary Document**) to reduce the existing heterogeneity in testing protocols across these countries, research studies and clinical settings. An obvious extension would be to devise a “global neuropsychological testing standard” that can accommodate the social, cultural, and economic diversity inherent in multi-continent research programmes. Although complex, such a pursuit is not wholly unrealistic. Many of the recommended protocols comprise measures that have been translated in languages prevalent in LMiC regions and could serve as a starting point for further validation. More recent exploratory measures that index functioning of AD-relevant neural correlates and which are less affected by or are independent of socio-cultural factors could provide suitable surrogates of early disease onset and progression.

## CHALLENGES AND OPPORTUNITIES FOR COGNITIVE ASSESSMENT OF AD IN LOW- AND MIDDLE-INCOME COUNTRIES

Major challenges in promoting AD research in LMiC regions have been suggested in detail elsewhere (ref. s22 in **Online Supplementary Document**). Our focus is on the important obstacle that is the lack of culturally appropriate assessment tools for the detection of cognitive change in the earliest stages of disease. Suitable normative data are generally not available (ref. s23 in **Online Supplementary Document**) and even when it is, it is not always appropriate for all members of that ethnic group (ref. s24 in **Online Supplementary Document**). There is still notable controversy surrounding the validity of adjusting neuropsychological test performance to account for ethnicity or regional background. Support for adjustments comes from studies which demonstrate poorer test specificity using White-normed reference data for minority samples living in western cultures relative to White samples. Consequently, greater number of false positives for cognitive impairment in minority groups are commonly noted (ref. s25 and s26 in **Online Supplementary Document**). On the other hand, the creation of ethnicity-based norms for cognitive test scores might conceal unidentified effects, such as genetic or lifestyle susceptibilities, that could contribute to group disparities in cognitive scores (ref. s27 in **Online Supplementary Document**). Regardless of one’s position within this debate, it should be recognized that the practice of simply translating measures developed in HiCs into alternative language versions dismisses the notable influence of local socio-cultural approaches to processing and deciphering information, above and beyond the influence of linguistic factors on test performance (ref. s28 in **Online Supplementary Document**). Many of these measures are weighted on language and verbal memory domain scores which are notoriously confounded by educational background and literacy levels (ref. s29 and s30 in **Online Supplementary Document**). Although, some research groups have made progress in validating existing cognitive tests in LMiC populations where wide variations in these variables exist (ref. s31 in **Online Supplementary Document**).

Parra (ref. s32 in **Online Supplementary Document**) suggests incorporating novel measures that show good specificity for early stages of AD and are insensitive to the effects of language and education altogether. One such task, the Visual Short-Term Memory Binding Test (VSTMBT) (ref. s34 in **Online Supplementary Document**), has been proposed as a gold standard test of early-stage AD assessment (ref. s21 in **Online Supplementary Document**). This task uses non-verbal stimuli, such as an array of shapes, to assess the ability to temporarily retain combinations of features (eg, form, colour) that make up complex objects from one presentation to the next. This task comprises little verbal information and simple instructions (eg, decide whether items are the same or different from those presented from the previous trial) making it accessible for people of low education and literacy level. Parra et al [s34] applied this task across the two culturally distinct AD patient groups: one from Colombia; the other from Scotland. The groups differed significantly in terms of language, education level and age (the Colombian group were hereditary early-onset AD, while the Scottish group were late-onset sporadic AD) but showed equivalent levels of impaired performance on the test. The task was able to differentiate performances of the AD groups from their respective healthy control groups. A lack of age effect was also demonstrated when the two control groups, differing significantly in mean age, were compared. These results indicate that the VSTMBT is able to isolate the effects of AD pathology from ‘normal ageing’ as well as culture, language and education. This is a crucial factor as, among older people, illiteracy remains high in less developed regions. Furthermore, in another study by the same research group, impaired performance on the VSTMBT was specific to individuals with AD and not for those with frontotemporal dementia, vascular dementia, lewy body dementia and Parkinson disease dementia (ref. s35 in **Online Supplementary Document**). The VSTMBT may therefore be an inexpensive non-invasive candidate for detecting early AD-specific cognitive change, and possibly early underlying disease, rather than a general screen for cognitive impairment associated with a range of dementias.

Like the VSTMBT, many novel cognitive tasks have been developed to isolate functioning of specific areas relevant to the AD disease process, such as the hippocampal formation, where amyloid and tau deposition may initially occur (ref. s35 in **Online Supplementary Document**). There are recommendations to include these novel tasks in neuropsychological test batteries in clinical trials for AD (ref. s18 and s19 in **Online Supplementary Document**) which has further substantiated their potential value as sensitive tools for the earliest cognitive markers. Several of these recommendations center around detection of early decline in episodic memory, the capacity to remember personally experienced events in space and time (ref. s36 in **Online Supplementary Document**). Verbal episodic memory is usually assessed through word lists recall and short vignettes; restricting use to literate populations. Moreover, since the information to be recalled is specific to the artificial experimental setting, their real-world applicability has been criticized (ref. s37 in **Online Supplementary Document**). Visually-based ecological episodic memory tasks exist, such as the Three Objects-Three Places screening task (ref. s38 in **Online Supplementary Document**) and might overcome these limitations. In this simple task, an experimenter hides three objects in a testing room in full view of the patient. The patient is then asked to recall the location of the hidden objects after some delay. The task has shown high sensitivity in prodromal and diagnosed AD but whether it is able to detect preclinical stages of AD remains to be seen. Another task that might show comparable real-world applicability and is sensitive to preclinical AD is the Face-Name association task (ref. s39 and s40 in **Online Supplementary Document**). Participants are presented with faces alongside information, such a name or occupation, and asked to recall this detail in a later trials when only the face is presented. The task simulates everyday memory exercises of greeting and recalling information about unfamiliar people. The paradigm can be easily and cost-effectively adapted to suit LMiCs regions, with faces, names or occupations that are relevant to local communities. A validated Spanish version of the task already exists (ref. s41 in **Online Supplementary Document**) and a cross-cultural validation of a similar paradigm is under-development in India (ref. s42 in **Online Supplementary Document**).

Tasks that restrict or are independent of verbal information may represent a helpful step towards reducing the effects of language or literacy on test performance. However, it would be a mistake to assume that non-verbal tests are impervious to the effects of culture. For example, Aruarco community members from rural Colombia showed difficulties in remembering details of complex, but nonsense, figures from a visual memory test when compared to westernized-Colombians and Canadians, despite the researchers accounting for educational differences between groups. Performance on another visually-based task in which participants were tested on the recognition of superimposed objects, some of which were objects pervading the Aruaco environment, was at ceiling level in the Aruarco group (ref. s43 in **Online Supplementary Document**). The recollection of arbitrary drawings may not be a culturally-important or practiced exercise for the Aruarco people compared to more westernised societies; their lack of familiarity with such a task and its contents possibly influencing performance. It is therefore important that any measures that are purported to reduce bias in cross-cultural situations, are investigated for their robustness against non-verbal aspects of culture or are adapted to ensure administration is ecologically-valid for the population under investigation. Recent integration of technological developments with neuropsychology may assist in this regard.

## THE FUTURE OF NEUROPSYCHOLOGICAL RESEARCH IN LMICS

A related issue regarding neuropsychological testing in LMiCs are that they are heavily dependent on the training and competency of the assessor. However, unlike biomarker ascertainment, more recent cognitive tasks comprise simple administration and scoring schemes with minimal requirements for expertise or training (ref. s15 in **Online Supplementary Document**). Developed economies are increasingly abandoning pen and paper measures in favour of computerised tasks, a progress already adopted in recent LMiC validation studies (eg, ref. s44 in **Online Supplementary Document**). Advantages of computerized tests include reduced administrator participation, allowing for faster and less costly data collection alongside automated scoring schemes that mitigate interviewer bias. A growing uptake of mobile technologies, such as smart phones, across some LMiC regions could further improve data collection methods through real-time behavioural, functional and some physiological assessments over longer and more frequent time points (ref. s45 in **Online Supplementary Document**). Novel technologies also enable the use of virtual reality tasks, such as the Virtual Supermarket task (ref. s46 in **Online Supplementary Document**), in which participants navigate a route of several 90 degree turns through a simulated supermarket from the first-person perspective. The task assesses egocentric spatial processing (location of objects in relation to self), impairment of which may be an early cognitive marker of AD (ref. s47 in **Online Supplementary Document**). Virtual reality allows for greater ecological validity through the opportunity to model circumstances from everyday life and create testing environments that are applicable to regional contexts (such as local natural or city landmarks in a navigation task). In the above example, instead of a supermarket and a trolley, similar navigation routes can be created that fit the environment of the test-takers, such as a local market. The rehabilitative potential of these technologies has been considered (ref. s48 in **Online Supplementary Document**); however, institutional readiness for their adoption in health care settings in HiCs remains elusive, let alone in LMiCs.

## CONCLUSION

The World Health Organisation (WHO) has set out a public health response for dementia, which aims to “progress globally towards better prevention, diagnosis, treatment and care for people with dementia” (ref. s49 in **Online Supplementary Document**). The shift towards studying preclinical and prodromal forms of dementia to provide secondary prevention strategies is aligned with the WHO’s goals. Most research studies modelling risk prediction and dementia conversion in these populations are being conducted in HiCs, leaving LMiCs less able to directly benefit from novel disease insights or capitalise on technological advances and industry investment. Effectively, LMiCs are excluded from participating fully in this global agenda. Since the accurate assessment of cognitive outcomes is fundamental to these research and care programmes, a first step in this direction would be to advance the most sensitive, socially and culturally competent cognitive measures in these regions. It is important that these measures are also sensitive and specific proxies of disease onset and progression, especially for countries where biomarker capture is difficult or unrealistic. Some examples of candidate tasks have been presented here. Further work to establish their cultural-competence in non-western cultures is required, but their adoption may assist in modernizing cognitive ageing research studies across LMiC areas and allow for better research integration between developing and developed countries.

## Additional Material

Online Supplementary Document
