# Supplementary material for: Neuropsychological assessment in preclinical and prodromal Alzheimer disease: a global perspective
Source: J Glob Health. 2019 Apr 24;9(1):010317. doi: 10.7189/jogh.09.010317 (PMC6486120; doi:10.7189/jogh.09.010317)
Supplement: Online Supplementary Document [file jogh-09-010317-s001.pdf]

J Glob Health 2019; 9: 010317

Supplementary references

s11 Albert MS, DeKosky ST, Dickson D, Dubois B, Feldman HH, Fox NC, et al. The diagnosis of mild cognitive impairment due to Alzheimer's disease: recommendations from the National Institute on Aging-Alzheimer's Association workgroups on diagnostic guidelines for Alzheimer's disease. Alzheimers Dement. 2011;7:270-9.

s12 Jack CR, Jr., Bennett DA, Blennow K, Carrillo MC, Dunn B, Haeberlein SB, et al. NIA-AA Research Framework: Toward a biological definition of Alzheimer's disease. Alzheimers Dement. 2018;14:535-62.

s13 Fox NC, Black RS, Gilman S, Rossor MN, Griffith SG, Jenkins L, et al. Effects of Abeta immunization (AN1792) on MRI measures of cerebral volume in Alzheimer disease. Neurology. 2005;64:1563-72.

s14 Albert M, Zhu Y, Moghekar A, Mori S, Miller MI, Soldan A, et al. Predicting progression from normal cognition to mild cognitive impairment for individuals at 5 years. Brain. 2018;141:877-87.

s15 Logie RH, Parra MA, Della Sala S. From Cognitive Science to Dementia Assessment. Policy Insights Behav Brain Sci. 2015;2:81-91.

s16 Hoefeijzers SaC, Clara and Parra, Mario A. Is it time to change the way we detect Alzheimer's disease and monitor its progression? Towards affordable and theory-driven approaches from cognitive neurosciences. JSM Alzheimers Dis Relat Dement. 2016; 3 1028

s17 K Ritchie, M Ropacki, B Albala, J Harrison, J Kaye, J Kramer, C Randolph, CW Ritchie. Recommended cognitive outcomes in preclinical Alzheimer's disease: Consensus statement from the European Prevention of Alzheimer's Dementia project. Alzheimers Dement. 2017 Feb;13(2):186-195. doi: 10.1016/j.jalz.2016.07.154.

s18 DM Rentz, MA Parra Rodriguez, R Amariglio, Y Stern, R Sperling and S Ferris. Promising developments in neuropsychological approaches for the detection of preclinical Alzheimer's disease: a selective review. Alzheimers Res Ther. 2013 Nov 21;5(6):58. doi: 10.1186/alzrt222. eCollection 2013.

s19 Donohue MC, Sperling RA, Salmon DP, Rentz DM, Raman R, Thomas RG, et al. The preclinical Alzheimer cognitive composite: measuring amyloid-related decline. JAMA Neurol. 2014;71:961-70.

s20 Paulino Ramirez Diaz S, Gil Gregório P, Manuel Ribera Casado J, Reynish E, Jean Ousset P, Vellas B, et al. The need for a consensus in the use of assessment tools for Alzheimer's disease: the Feasibility Study (assessment tools for dementia in Alzheimer Centres across Europe), a European Alzheimer's Disease Consortium's (EADC) survey. Int J Geriatr Psychiatry. 2005;20:744-8

s21 Costa, T Bak, P Caffarra, C Caltagirone, M Ceccaldi, F Collette, et al. The need for harmonisation and innovation of neuropsychological assessment in neurodegenerative

dementias in Europe: consensus document of the Joint Program for Neurodegenerative Diseases Working Group. Alzheimers Res Ther. 2017; 9:27. https://doi.org/10.1186/s13195-017-0254-x

s22 MA Parra. Overcoming barriers in cognitive assessment of Alzheimer's disease. Dement Neuropsychol. 2014;8(2):95-98.

S23 J Manly. Advantages and Disadvantages of Separate Norms for African Americans. Clin Neuropsychol, 2005; 19(2): 270-275. doi: 10.1080/13854040590945346

s24 M Rivera Mindt, D Byrd , P Saez & J Manly. Increasing Culturally Competent Neuropsychological Services for Ethnic Minority Populations: A Call to Action. Clin Neuropsychol; 2009; 24(3): 429-453

s25 Fillenbaum G, Heyman A, Williams K, Prosnitz B, Burchett B. Sensitivity and specificity of standardized screens of cognitive impairment and dementia among elderly black and white community residents. J Clin Epidemiol. 1990;43:651-60.

s26 Gurland BJ, Wilder DE, Cross P, Teresi J, Barrett VW. Screening scales for dementia: Toward reconciliation of conflicting cross-cultural findings. Int J Geriatr Psychiatry. 1992;7:105-13.

s27 Manly JJ, Echemendia RJ. Race-specific norms: using the model of hypertension to understand issues of race, culture, and education in neuropsychology. Arch Clin Neuropsychol. 2007;22:319-25.

s28 Altarriba J. The Influence of Culture on Cognitive Processes. In: Altarriba J, editor. Advances in Psychology. 103: North-Holland; 1993. p. 379-84.

s29 SE O'Bryant, JD Humphreys, GE Smith, RJ Ivnik, NR Graff-Radford. RC Petersen et al. Detecting dementia with the mini-mental state examination in highly educated individuals. Arch Neurol. 2008;65(7):963-7. doi: [10.1001/archneur.65.7.963]

s30 A Ardila, PH Bertolucci, LW Braga, A Castro-Caldas, TJudd, MH Kosmidis, et al. Illiteracy: The Neuropsychology of Cognition Without Reading, Arch Clin Neuropsychol, 2010; 25(8):689–712

s31 AT Jotheeswaran, JD Williams, MJ Prince. The predictive validity of the 10/66 dementia diagnosis in Chennai, India: a 3-year follow-up study of cases identified at baseline. Alzheimer Dis Assoc Disord. 2010;24(3):296-302.

s32 M Prince, D Acosta, H Chiu, M Scazufca and M Varghese. Dementia diagnosis in developing countries: A cross-cultural validation study. Lancet, 2003; 361(9361): 909-917.

s33 MA Parra. Overcoming barriers in cognitive assessment of Alzheimer's disease. Dement Neuropsychol. 2014;8(2):95-98.

s34 MA Parra, SD Sala, A Abrahams, RH Logie, LG Mendez, F Lopera. Specific deficit of colour-colour short-term memory binding in sporadic and familial Alzheimer’s disease. Neuropsychologia. 2011;49:1943-1952

s35 Della Sala S, Parra MA, Fabi K, Luzzi S, Abrahams S. Short-term memory binding is impaired in AD but not in non-AD dementias. Neuropsychologia. 2012;50:833-40

s36 E Tulving. Elements of episodic memory, Clarendon Press, Oxford. 1983.

s37 F Newcombe andL Artiola i Fortuny. Problems and perspectives in the evaluation of psychological deficits after cerebral lesions. Int Rehabil Med. 2009; 1(4): 182-192. Doi: https://doi.org/10.3109/03790797909164041

s38 A Prestia, R Rossi, C Geroldi, S Galluzzi, M Ettori, G Alaimo, G., et al. Validation study of the three-objects-three-places test: A screening test for Alzheimer's disease. Exp Aging Res. 2006; 32:395–410. doi:10.1080/03610730600875767.

s39 Rentz DM, Amariglio RE, Becker JA, Frey M, Olson LE, Frishe K, et al. Face-name associative memory performance is related to amyloid burden in normal elderly. Neuropsychologia. 2011;49:2776-83.

s40 Sperling RA, Laviolette PS, O'Keefe K, O'Brien J, Rentz DM, Pihlajamaki M, et al. Amyloid deposition is associated with impaired default network function in older persons without dementia. Neuron. 2009;63:178-88

s41 Alegret M, Valero S, Ortega G, Espinosa A, Sanabria A, Hernández I, et al. Validation of the Spanish Version of the Face Name Associative Memory Exam (S-FNAME) in Cognitively Normal Older Individuals. Arch Clin Neuropsychol. 2015;30:712-20.

s42 Lukose A, Kv R, N Rao G, Palanimuthu Thangaraju S, Ritchie K, Rao N. Cross-cultural adaption of the Computerized Assessment of Information Processing battery (COGNITO) for an Indian longitudinal study on rural elderly: An elucidation of the adaptation process. Alzheimer’s Association International Conference® (AAIC®) Satellite Symposium, 18-19 December 2018, Bengaluru, India

s43 Ardila A, Moreno S. Neuropsychological test performance in Aruaco Indians: an exploratory study. J Int Neuropsychol Soc. 2001;7:510-5.

s44 GW Humphreys, MD Duta, L Montana, N Demeyere, C McCrory, J Rohr et al. Cognitive Function in Low-Income and Low-Literacy Settings: Validation of the Tablet-Based Oxford Cognitive Screen in the Health and Aging in Africa: A Longitudinal Study of an INDEPTH Community in South Africa (HAALSI). J Gerontol B Psychol Sci Soc Sci. 2016;72(1):38-50.

s45 PA Areàn, LK Hoa, G Andersson. Mobile technology for mental health assessment. Dialogues Clin Neurosci. 2016;18(2):163-9.

s46 S Tu, S Wong, JR Hodges, M Irish, O Piguet, M Hornberger. Lost in spatial translation. A novel tool to objectively assess spatial disorientation in Alzheimer's disease and frontotemporal dementia. Cortex. 2015; 67:83-94

s47 S Serino, P Cipresso, F Morganti, G Riva. The role of egocentric and allocentric abilities in Alzheimer's disease: a systematic review. Ageing Res. Rev. 2014; 16: 32-44.

s48 AA Rizzo, JG Buckwalter and U Neumann. Virtual reality and cognitive rehabilitation: A brief review of the future. J Head Trauma Rehabil; 1997, 12(6): 1-15. http://dx.doi.org/10.1097/00001199-199712000-00002

s49 Draft global action plan on the public health response to dementia. Report by the Director-General, World Health Organisation; 2017.

(http://apps.who.int/gb/ebwha/pdf_files/EB140/B140_28-en.pdf?ua=1, accessed 5 September 2018).
